# Intracranial Solitary Fibrous Tumor

**DOI:** 10.5334/jbr-btr.1213

**Published:** 2017-02-20

**Authors:** Eveline Claus, Patrick Seynaeve, Jeroen Ceuppens, Alain Vanneste, Koenraad Verstraete

**Affiliations:** 1 UZ Gent, BE; 2 AZ Groeninge Kortrijk, BE

**Keywords:** Intracranial, Solitary, Fibrous, Tumour

## Abstract

Solitary fibrous tumours are rare mesenchymal spindle-cell tumours that occur most often in the visceral pleura or liver. If they occur intracranially, they are extra-axially located and develop from the meninges. In those cases, the differential diagnosis has to be made with other intracranial extra-axial-located tumours, such as meningeoma and hemangiopericytoma. We report a 32-year-old woman with an intracranial solitary fibrous tumour and review the latest literature regarding the imaging characteristics of this tumour.

## Introduction

Solitary fibrous tumours are rare spindle-cell mesenchymal tumours, which rarely occur in the central nervous system [1,3]. When they do occur intracranially, they are usually extra-axially located [1]. The imaging characteristics of these tumours have been scarcely reported and mostly as solitary case reports [1]. Pre-operative definitive diagnosis is difficult due to the atypical imaging characteristics, but the differential diagnosis can be limited by imaging. In this case report, we discuss the differential diagnosis of extra-axial intracranial masses and how to distinguish them based on imaging characteristics. Furthermore, a review of the literature regarding imaging characteristics of intracranial solitary fibrous tumours was performed.

## Case Report

A 32-year-old patient presented at the emergency department with paresthesia in the left arm that migrated to the left paravertebral region and the left leg. Her left arm also felt heavy. The episode had lasted for less than a minute. The patient was a smoker with an unremarkable medical history.

Clinical neurological examination was negative, as were the hematological and biochemical results. The electroencephalogram (EEG) showed deceleration in the right hemisphere, but no other abnormalities.

**Figure 1:**
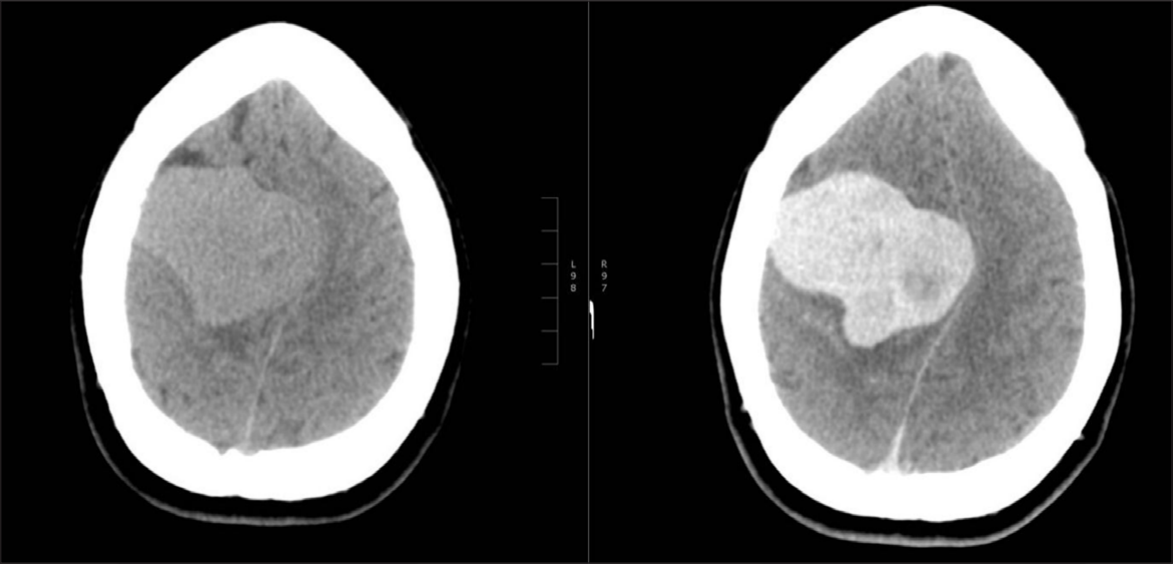
Plain CT and contrast-enhanced CT (CECT) show a lobulated, heterogeneously enhancing mass with several non-enhancing areas.

The computed tomography (CT) scan showed a large, sharply delineated, lobulated mass in the right hemisphere (Figure 1). The mass had a broad dural base. There was minimal surrounding edema. The tumour showed intense heterogeneous contrast enhancement around small, centrally located non-enhancing areas. There was mass effect with midline shift, but no subfalcine herniation.

**Figure 2:**
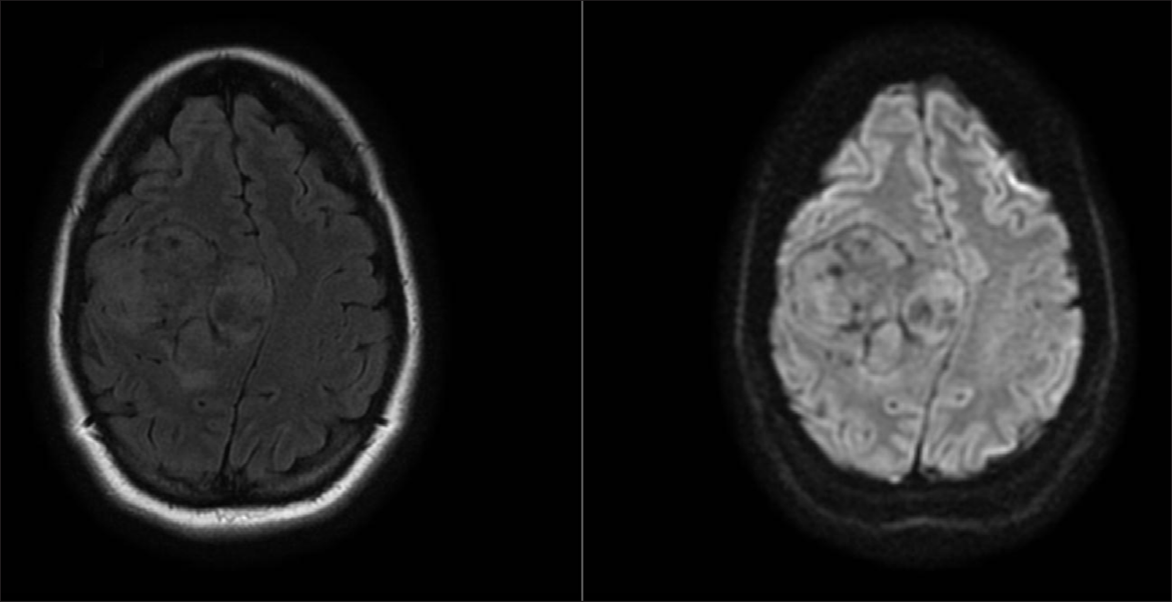
T2FLAIR and DWI show a midline shift but no diffusion restriction.

**Figure 3A and B:**
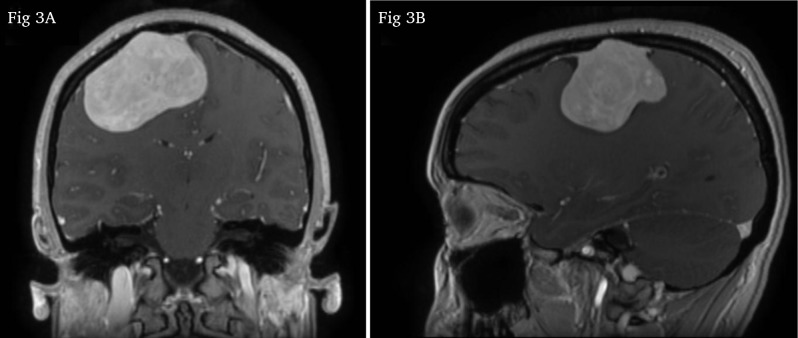
Coronal and sagittal T1-weighted images after intravenous administration of gadolinium contrast clearly demonstrate the dural tail sign.

**Figure 4:**
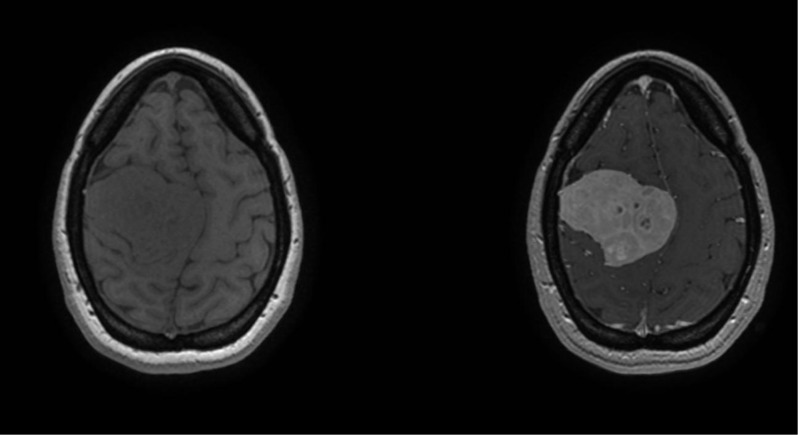
T1-weighted images before and after intravenous administration of gadolinium contrast show heterogeneous contrast enhancement with less enhancement of the central areas.

The magnetic resonance imaging (MRI) scan two days later showed a heterogeneous mass which was isointense on T1-weighted and hyperintense on T2-weighted sequences (compared to grey matter Figure 2). After administration of gadolinium contrast, there was intense heterogeneous enhancement (Figure 3A and B; Figure 4). The MRI scan did not show other suspicious lesions. Diffusion-weighted imaging (DWI) did not reveal areas with diffusion restriction (Figure 2). MRI spectroscopy showed a high choline- and myo-inositol peak in both short and long echo time series.

**Figure 5:**
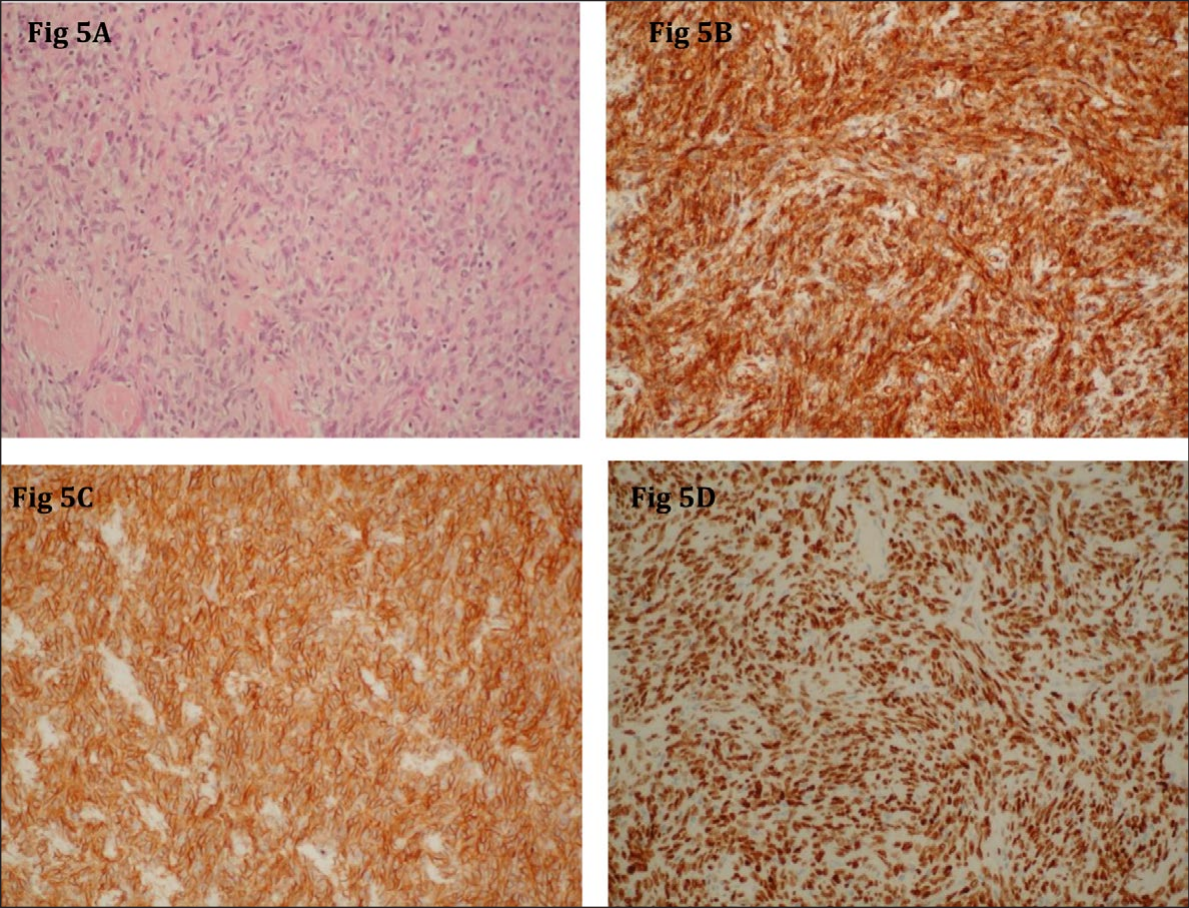
Hematoxylin-eosin stain with ×200 magnification **(A)** shows a high concentration of cells. CD 34 stain with ×200 magnification is positive **(B)**. CD 99 stain (×200) **(C)**, and STAT-6 stain (×200) **(D)** confirm the positive result.

The lesion was resected. Histologic examination showed a highly cellular tumour with multiple spindle cells as well as focal areas of small cells with elevated cytoplasmatic index (Figure 5A). Immunohistochemistry showed a diffuse, strongly positive CD34 stain (Figure 5B) and variably positive Ki-67 as well as strongly positive CD 99 (Figure 5C) and STAT-6 staining (Figure 5D). The diagnosis of an intracranial solitary fibrous tumour was made.

The tumour was completely resected, without need for further treatment. The patient was scheduled for follow-up MRI after three months.

## Differential Diagnosis

Based on the presentation of the tumour, other extra-axial intracranial tumours should be considered in the differential diagnosis. They include meningeoma, hemangiopericytoma, dural metastasis, dural lymphoma, isolated neurosarcoidosis, and gliosarcoma.

A meningeoma is an extra-axial tumour arising from the arachnoïdale cells of the meninges. On CT, a meningeoma appears as a sharply delineated hyperdense mass with intense homogeneous contrast enhancement. On MRI, it is slightly hypo- to isointense compared to grey matter on T1-weighted images with homogeneous contrast enhancement. The intensity on T2-weighted imaging is variable. In contrast to SFT, a dural tail sign and calcification of the tumour can frequently be observed. A dural tail sign is not specific to any extra-axial tumour, since meningeomas, hemangiopericytomas, and ISFTs have all been reported showing a dural tail sign. The adjacent bone may help to differentiate meningeoma from ISFT. The adjacent bone is often thickened in meningeomas; SFT, in contrast, can erode the adjacent bone [1,2].

Hemangiopericytoma (HPC) is a mesenchymal tumour that is considered identical to SFT when it is located extracranially [3,4]. Intracranially, however, both tumours exhibit very distinct biological characteristics. HPC is considered a cellular, more aggressive type of SFT. The more benign ‘fibrous’ variant has been termed ‘the fibrous type SFT’ [3,4]. It is hard to distinguish HPC from SFT on imaging. Making the distinction is important, however, since HPCs show a high rate of recurrence and late extracranial metastasis compared to SFTs.

Dural metastasis often presents as an enhancing biconvex mass displacing the brain. The most common neoplasms to develop dural metastasis are breast, lung, and prostate cancer. Dural metastasis from melanoma, lymphoma, renal cell carcinoma, and gastric carcinoma can also occur but are less frequent [5]. Dural metastases are frequently solitary, which can lead to misdiagnosis. The skull bone is frequently involved. The primary tumour determines the imaging characteristics. Most dural metastases are T1-hypointense and T2-hyperintense compared to grey matter and show diffusion restriction due to high cellularity.

Primary dural lymphoma is a rare dural neoplasm. The majority represents low-grade mucosa-associated lymphoid tissue (MALT) [5]. The pathogenesis of lymphoid tissue is still unclear. A dural lymphoma is typically seen as a hyperdense mass on CT, reflecting the high cellularity of the tumour [5]. On MRI, it is iso-to hypointense compared to grey matter on both T1- and T2-weighted images. It shows diffuse homogeneous enhancement after contrast administration.

Isolated neurosarcoidosis without systemic manifestation is very rare. It has very indistinct imaging features, which makes it difficult to diagnose. Neurosarcoidosis presents itself as multiple heterogeneous meningeal lesions on MRI. It tends to be isointense on T1-weighted images [5]. T1-weighted images after the administration of gadolinium contrast show nodular and linear enhancement. On T2-weighted imaging, the hypointense areas correlate with fibrocollageneous tissue, while the hyperintense areas represent inflammation. Dural lesions are rare and are often accompanied with other intracranial lesions, typically located in the basal cisterns (e.g. optic chiasm, infundible, hypothalamus, cranial nerves).

Gliosarcoma is a rare variant of glioblastoma multiforme, with mesenchymal and glial elements. It presents as a heterogeneous enhancing mass, with focal areas of necrosis and hemorrhage. It is often accompanied by marked perilesional oedema. At MRI, it presents itself as a heterogeneous T1-hypointense mass with irregular rim enhancement, without enhancement of the central (necrotic/hemorrhagic) areas. It is surrounded by a T2-hyperintense area, representing surrounding edema.

## Discussion

Of the aforementioned differential diagnosis, some diagnoses can be excluded based on the imaging findings. Because of the absent alanine peak on the MR spectroscopy, a meningeoma can be ruled out. Dural metastasis is less likely because there is no diffusion restriction and no history of primary malignancy. A primary dural lymphoma is hypointense on T2-weighted imaging and not hyperintense. Gliosarcoma often shows prominent perilesional edema and focal areas of necrosis and hemorrhage, which makes the diagnosis less likely. Isolated neurosarcoidosis typically presents with multiple lesions and systemic manifestations, which makes this diagnosis unlikely, although it cannot be ruled out based on the imaging characteristics alone. An intracranial solitary fibrous tumour and a hemangiopericytoma are both compatible with the imaging characteristics. These tumours cannot be distinguished from one another through imaging. Histologic examination is necessary to provide the definitive diagnosis. The definitive diagnosis in this case was made by histologic examination, which showed a solitary fibrous tumour.

Solitary fibrous tumours are rare spindle-cell mesenchymal tumours, which only rarely occur intracranially [1,3]. They have been reported more frequently in the visceral pleura and liver, skin, orbits, and paranasal sinuses [1]. When they do occur intracranially, they usually develop from mesenchymal cells in the meninges and are extra-axially located [1]. The most frequent location is along the tentorium cerebelli, followed by the frontal convexity, cerebellopontine angle, ventricles, falx cerebri, and posterior fossa [2].

Symptoms associated with an ISFT are headache, gait disturbance and imbalance, weakness, visual loss, cranial nerve dysfunction, nausea/vomiting, and altered mental status or confusion [*2*].

Histological examination shows spindle cells in fascicular organisation with intercellular collagen bundles [1,6]. The tumour is further characterised by a positive CD34 stain and a positive STAT-6 stain [4,6]. Some authors also reported positive bcl-2 and vimentin staining in ISFTs [6,7]. Dedifferentiated SFT may be positive for p53, while the conventional, fibrous component is usually negative or shows scarcely scattered positive staining [4]. It is also associated with p16 deletion [4].

The imaging characteristics of ISFTs have been described by Clarençon et al. [1], based on a restrospective cohort of nine patients. The majority of these lesions were large, multi-lobular and well demarcated. Most were entirely solid, while a few showed cystic components. On CT, half the masses were heterogeneous. The homogeneous lesions were iso- (25%) or hyperdense, with calcifications in a minority of the cases [1,2]. Hypodense lesions have also been reported in a few cases [2]. In just more than half the cases, there was erosion of the skull adjacent to the tumour. There was intense enhancement after IV iodinated contrast injection. The reported enhancement patterns include homogeneous enhancement as well as partial or heterogeneous enhancement [2].

At MRI, a majority of cases are homogeneous on T1-weighted imaging (most often isointense). In literature, the most common presentation is a heterogeneous pattern on T2-weighted imaging with hypointense areas [1,7]. This may be explained by the existence of two separate components within the tumour. The first component is fibrosis, represented by T2-hypointense areas that show intense contrast enhancement. The presence of this component in a meningeal mass suggests SFT.

The second hypercellular component presents as a T2 iso- or hyperintense area with moderate heterogeneous enhancement [1,2]. The combination of these two components gives rise to the so-called yin yang sign, which is associated with ISFTs. Other reports [2,7] state that a hypointense or a hyperintense T2 signal is more common than a heterogeneous T2 signal, with heterogeneous contrast enhancement in most cases. In contrast to meningeomas, ISFTs rarely demonstrate contrast enhancement of the adjacent meninges (dural tail sign). Significant peritumoral edema is seen in a minority of cases (25%).

In some cases, diffusion-weighted imaging was performed. The cystic components of a multi-cystic SFT showed an elevated apparent diffusion coefficient (ADC), as expected from the high fluid content (T2 shine through) [1]. Solid SFTs showed areas with restricted diffusion on ADC maps [1]. There is overlap with the diffusivity of meningeomas. Current evidence has only limited information about MR spectroscopy in SFTs, with just one patient who underwent MR spectroscopy [1]. This showed an elevated choline/creatinin ratio and a myoinositol peak. Literature states that there are no lactate nor alanine peaks.

Perfusion MRI usually shows an increase of the cerebral blood volume (7–7.5 fold rCBV compared to the cerebral blood volume of the contralateral white matter) [1]. The time to peak and mean transit time is lengthened.

There is currently no guideline regarding the optimal therapeutic approach for SFTs. Total excision of the mass is clearly superior to subtotal resection, with a 16-fold increase in recurrence ratio with subtotal resection (without adjuvant therapy), compared to total resection. Pre-operative embolization, pre-operative chemotherapy and post-operative adjuvant radiotherapy have been performed. No conclusion can currently be drawn regarding the effectiveness of these measures. There is no optimal chemotherapeutic regimen for SFTs yet either.
